# Effects of intravenous lipid emulsions on Jurkat cells assessed using label-free deformability cytometry

**DOI:** 10.1038/s41598-025-33582-7

**Published:** 2025-12-30

**Authors:** Lija Fajdiga Lebar, Jernej Repas, Bor Ivanuš, Darin Lah, Nina Bernat, Lara Betocchi, Miran Bürmen, Špela Zemljič, Jure Derganc

**Affiliations:** 1 Faculty of Medicine, Institute of Biophysics, University of Ljubljana, Tržaška Cesta 25, 1000 Ljubljana, Slovenia; 2 Faculty of Electrical Engineering, University of Ljubljana, Vrazov Trg 2, 1000 Ljubljana, Slovenia

**Keywords:** Deformability cytometry, Lipid droplets, SMOFlipid, Omegaven, Parenteral lipid emulsions, Deep neural network classification, Biochemistry, Biological techniques, Biotechnology, Drug discovery, Immunology

## Abstract

Intravenous lipid emulsions (ILEs) are routinely used in clinical nutritional support and exert immunomodulatory effects, although they remain poorly understood. This is particularly relevant for cell-based immunotherapies, such as CAR-T therapy, where interactions between immune cells and circulating lipids may influence treatment efficacy. We investigated the effects of two commonly used ILEs (SMOFlipid and Omegaven) on Jurkat cells, using deformability cytometry (DC). Beyond assessing cell mechanics, we extended DC with two novel, label-free approaches: deep neural network–based image classification for cell state evaluation and morphological analysis for lipid droplet detection.

Our results show that ILE composition strongly affects cytotoxicity. Omegaven, rich in omega-3 polyunsaturated fatty acids, was markedly more toxic than SMOFlipid, which mainly contains omega-6 and omega-9 fatty acids. While cell stiffness remained largely unchanged, DC effectively detected reduced viability and increased lipid droplet accumulation. Seahorse metabolic analysis supported these findings, showing no significant changes in energy metabolism, suggesting that excess lipids were stored rather than metabolized.

This study highlights a novel application of DC for high-throughput, label-free characterization of immune cells, providing insights into how ILEs may influence T cell biology and potentially impact the safety and efficacy of immunotherapies.

**Supplementary Information:**

The online version contains supplementary material available at 10.1038/s41598-025-33582-7.

## Introduction

Intravenous lipid emulsions (ILEs) are an indispensable component of parenteral nutrition, a life-saving therapy for individuals with impaired gastrointestinal function who cannot tolerate oral nutrition for prolonged periods. ILEs provide a source of non-protein energy and essential fatty acids (FAs), and they are administered both in critical care settings and as long-term therapy in home settings. Historically, the first ILEs used in clinical practice were based on soybean oil (SO), which is composed primarily of omega-9 and omega-6 fatty acids. However, their use was linked to several side effects, including parenteral nutrition–associated liver disease (PNALD). To address these concerns, alternative formulations were developed, such as olive oil (OO) emulsions, which demonstrated improved functionality and were associated with fewer adverse effects than SO^1^. The most commonly used ILEs today are fish oil (FO)-based ILEs (comprising omega-3 FAs) and balanced ILEs (omega-9, omega-6 and omega-3 FAs)^2^.

It has since been recognized that different ILEs exert diverse and complex immunomodulatory effects^3^. For instance, clinical studies have demonstrated that SO-based ILEs increase monocyte adhesion to endothelial cells and enhance lipopolysaccharide-stimulated production of pro-inflammatory cytokines in neutrophils and monocytes, while FO-based ILEs exhibit the opposite effects^4^. FO-containing emulsions have also been associated with reduced incidence of infections in critically ill patients^5,6^. The distinct immunomodulatory effects of different fatty acids have also been observed in cell lines and primary cell cultures in vitro. For instance, oleic acid (OA, omega-9) has been shown to be significantly less cytotoxic than linoleic acid (LA, omega-6) in lymphocytes T^7^ and in Jurkat cells^8,9^, which are a widely used model for human lymphocytes.

The immunomodulatory effects of ILEs are particularly important for patients for whom immune function is critical to the treatment outcomes, e.g., for cancer patients^10^. The functional state of the immune system is especially crucial in modern cellular immunotherapies, such as chimeric antigen receptor T cell (CAR-T) therapy^11^. In this therapeutic approach, lymphocytes are collected from the patient, genetically engineered, expanded ex vivo, and reinfused to target malignant cells. The functional fitness of CAR-T cells is essential for therapeutic efficacy^12^. Consequently, monitoring lymphocyte viability and fitness—typically via flow cytometry using fluorescently labelled antibodies—^13^ is a critical part of the treatment process. New biomarkers are continuously being identified as indicators of cell health, with increasing attention being given to biophysical markers^14,15^. The mechanical properties of immune cells, such as stiffness and deformability, are emerging as important determinants of immune cell function, including migration and extravasation^16^.

**Fig. 1:**
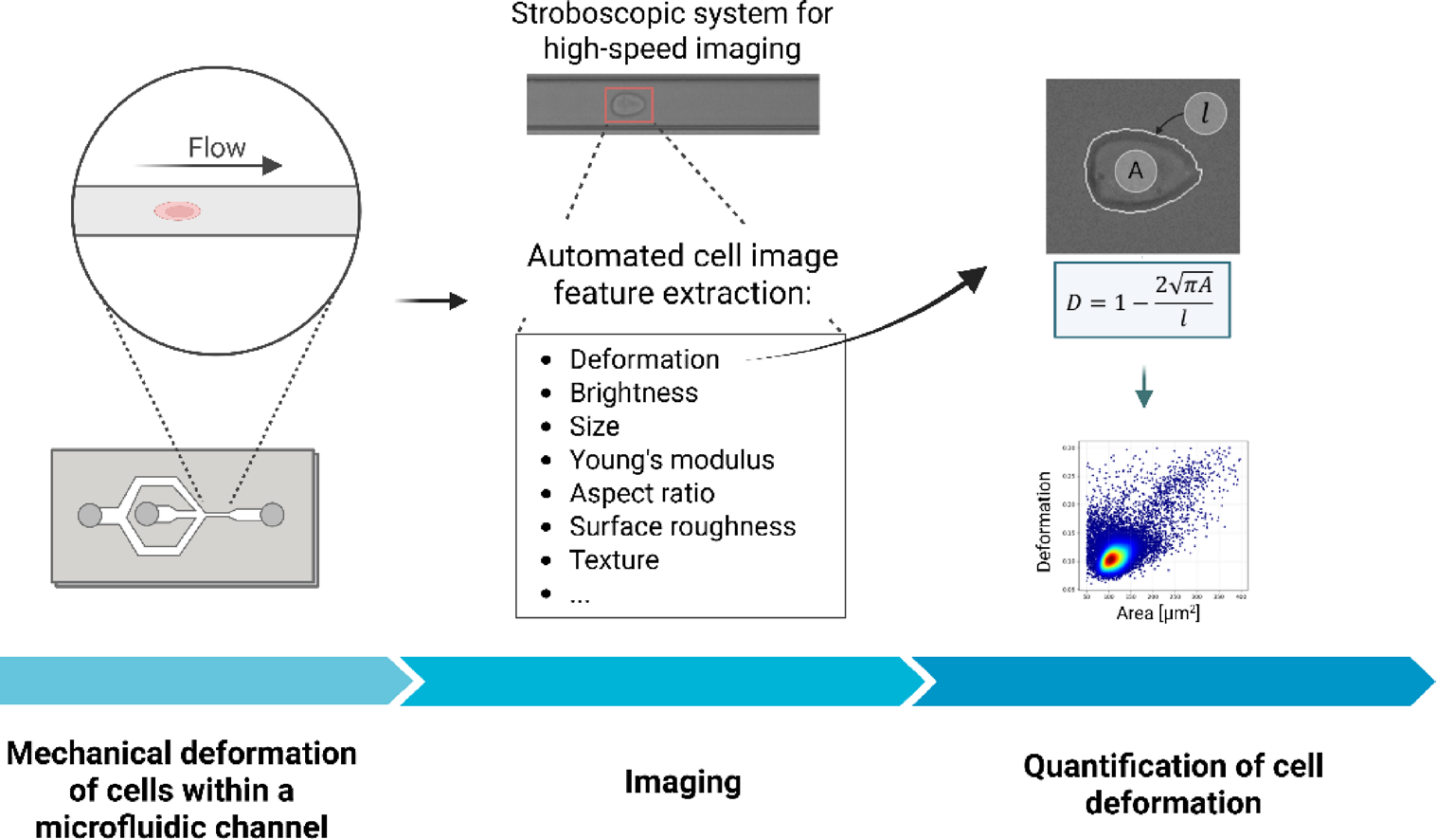
Principle of deformability cytometry. A schematic overview of the deformability cytometry method. At the core of this technique is imaging of cells as they flow through a narrow microfluidic channel, where high-viscosity buffer-induced shear forces cause them to deform. Imaging is followed by image segmentation and extraction of morphological features such as cell deformation, which is a parameter related to the ratio between the square root of the cell’s projected area and the perimeter of its contour. The method can measure up to 1000 cells per second, and the results are presented in the form of scatter plots showing the relationship between the measured parameters for individual cells, similarly to classical flow cytometry. Created in BioRender (https://BioRender.com/62h1rag).

Recently, deformability cytometry (DC) has emerged as a promising high-throughput method for imaging individual cells and measuring cell deformability^17,18^. This technique relies on brightfield imaging of individual cells as they are deformed by shear forces in rapid hydrodynamic flow through a narrow microfluidic channel. This enables the quantification of cell deformation alongside a range of morphological features (Fig. 1). For instance, DC has been used to classify blood cells into distinct subtypes, in a manner similar to classical complete blood count (CBC) analysis^19^. In many respects, DC is comparable to CBC and flow cytometry^20^; however, it additionally provides information on a novel mechanical biomarker—cell deformability—and operates without the need for fluorescent markers, making it a label-free technique.

In this study, we present the first DC of Jurkat cells incubated in two widely used clinical ILEs: Omegaven, a fish oil–based emulsion, and SMOFlipid, a balanced formulation. We found that both emulsions had only a minor effect on cell deformability; however, both exhibited cytotoxicity and led to intracellular lipid droplet accumulation at clinically relevant concentrations. Notably, Omegaven induced these effects at concentrations approximately ten times lower than SMOFlipid. The study provides a foundation for future investigations into primary lymphocytes, which are essential for ensuring the safe use of ILEs in patients undergoing immunotherapies.

## Materials and methods

### Cell culture and treatment

Jurkat cells (clone E6-1, ATCC, lot number: 70053340) were cultured in RPMI-1640 medium (Gibco, Life Technologies Limited, UK) supplemented with 10% fetal bovine serum (Gibco, Life Technologies Limited, UK) and 1% penicillin–streptomycin (Gibco, Life Technologies Limited, UK). Cells were maintained at 37 °C in a humidified incubator with 5% CO₂ and routinely split every 2–3 days to maintain concentrations below 1 × 10⁶ cells/mL. For experiments, cells were seeded at a concentration of 2.5 × 10^5^ cells/mL and incubated for 48 h before analysis.

Jurkat cells were treated with SMOFlipid and Omegaven ILEs diluted in RPMI-1640 medium to final lipid concentrations of 0.01, 0.05, 0.1, 0.5, and 1 mg/mL for SMOFlipid (Fresenius Kabi Deutschland GmbH, Bad Homburg, Germany), and 0.001, 0.005, 0.01, 0.05, and 0.1 mg/mL for Omegaven (Fresenius Kabi Austria GmbH, Graz, Austria). Cells were incubated with the ILEs for 48 h under standard culture conditions (37 °C, 5% CO₂).

For individual fatty acid treatment Jurkat cells were treated with four different free fatty acids: eicosapentaenoic acid (EPA) (Sigma-Aldrich, St. Louis, USA), docosahexaenoic acid (DHA) (Sigma-Aldrich, St. Louis, USA), oleic acid (OA) (Sigma-Aldrich, St. Louis, USA), and linoleic acid (LA) (Sigma-Aldrich, St. Louis, USA). The fatty acids were suspended directly in RPMI-1640 medium and added to the cells at final concentrations of 0.01, 0.05, 0.1, and 0.5 mg/mL. The cells were seeded at a density of 2.5 × 10^5^ cells/mL and incubated with the fatty acids for 48 h under standard culture conditions (37 °C, 5% CO₂).

### Activation of Jurkat cells

To activate the cells, the flat-bottomed cell culture plates were coated with PBS containing 10 µg/mL of anti-CD3 antibody (BioLegend, San Diego, CA, USA) for at least 2 h at 37 °C. After coating, the plates were washed three times with PBS. Jurkat cells were then added at a density of 2.5 × 10^5^ cells/mL. Anti-CD28 antibody (BioLegend, San Diego, CA, USA) was added to the cell suspension at a concentration of 5 µg/mL. The cells were then incubated for 48 h at 37 °C and 5% CO₂ to allow full activation. To confirm activation, CD69 surface expression was measured via immunofluorescence and analyzed using flow cytometry (see Supplementary Figure S1).

For ILE treatment of activated cells, the antibodies were washed away, and the cells were seeded in RPMI-1640 medium with ILEs, as described in the previous paragraph.

### MTS assay for cell viability

Jurkat cell viability was assessed after 48 h of incubation with ILEs using the CellTiter 96 AQueous One Solution Reagent (Promega, Madison, WI, USA), which contains the MTS tetrazolium compound and phenazine ethosulphate as an electron coupling reagent. The assay was conducted according to the manufacturer’s guidelines. In each well of a 96-well plate, 20 µL of the MTS reagent was added to 100 µL of the cell suspension. After an incubation period of 3–4 h at 37 °C with 5% CO₂, the absorbance was measured at 490 nm using a 96-well plate reader (Bio-Tek Instruments Inc., Winooski, VT, USA). Absorbance readings reflect the formation of soluble formazan, which forms in direct correlation with the number of viable cells. Cell viability was determined by calculating the ratio of the absorbance at 490 nm of the treated cells to that of the control cells.

### Deformability cytometry measurements

The mechanical properties of Jurkat cells were assessed using a custom-built DC system^21^. This setup included a stroboscopic illumination unit and an industrial camera (Ximea MC023MG-SY-UB, equipped with a Sony IMX-174 sensor) mounted on an inverted microscope (Nikon TE2000) with a 40 × objective. The cells were imaged with 0.5 μs light flashes at a rate of over 600 frames per second in 8-bit format.

Prior to measurement, the cells were centrifuged and resuspended in a buffer solution containing 0.6% methylcellulose (Buffer B, Zellmechanik Dresden GmbH, Germany) to increase viscosity and enhance deformation sensitivity^22^. The cell suspensions were drawn into syringes and introduced into the microfluidic system. Flow was controlled using Nemesys Syringe Pumps (Cetoni GmbH, Germany), at flow rates of 0.04 μL/s for the sample and 0.12 μL/s for the sheath fluid. Measurements were performed using FLIC20 microfluidic chips (Zellmechanik Dresden GmbH, Germany), which induce deformation of the cells as they pass through a constriction.

Bright-field images were acquired and analyzed using a Python script that included background subtraction, filtering, thresholding, and morphological operations to enable robust cell detection and segmentation (the script is available at https://github.com/biophysics-ul/rtdc-toolbox). From the segmented cell contours deformation was computed based on the contour perimeter (l) and projected surface area (A) according to the method described by^17^:

$$D = 1 - \frac{{2\sqrt {\pi A} }}{l}$$

We validated the performance of our DC system by measuring cell deformation across a range of flow rates (Supplementary Figure S2). As expected, deformation increased with higher flow rates, consistent with previous observations and theoretical predictions^17^. In a typical experiment, between 1,000 and 50,000 cells were measured, with a median of approximately 9,000 cells per experiment.

The density plot shown in Fig. 4 was generated using CytoPlot (version 2.21, Rivercyte GmbH, Erlangen, Germany).

### LipidTOX staining of lipid droplets for confocal microscopy and flow cytometry

To visualize intracellular lipid accumulation, the cells were stained using the HCS LipidTOX Green Neutral Lipid Stain (Invitrogen, Thermo Fisher Scientific, Waltham, MA, USA). First, Jurkat cells were first fixed by centrifugation (300 × g, 5 min) and resuspension in 4% paraformaldehyde in PBS, followed by a 30-min incubation at room temperature. After fixation, the cells were washed twice with PBS at room temperature. For staining, the LipidTOX dye was diluted in PBS and added to the cell suspension at a final concentration of 1:1000, followed by a 45-min incubation at room temperature. Finally, the cells were centrifuged (300 × g, 5 min) and resuspended in PBS for imaging and flow cytometry.

For confocal microscopy Nikon ECLIPSE TE2000-E microscope (Plan Apo TIRF objective, magnification 60 × , NA = 1.45) in the confocal mode (Nikon C1) was used.

To quantify lipid accumulation in Jurkat cells, we used the HCS LipidTOX Green Neutral Lipid Stain (Invitrogen) and performed flow cytometry analysis on an Attune NxT Flow Cytometer (Thermo Fisher Scientific, Waltham, MA, USA). A gating strategy was applied to select single cells based on forward and side scatter characteristics. The fluorescence-positive populations were determined using manually set gates, which were adjusted according to the signal of the unstained control samples.

### Training of deep neural network for classification of cell images

We developed a supervised machine-learning classifier using a convolutional neural network based on the ResNet18 architecture, due to its efficiency on mid-range hardware. The network architecture was customized by removing the final pooling and fully connected layers from the standard ResNet18 model and replacing them with a custom classifier composed of fully connected layers, ReLU activation, and dropout layers to reduce overfitting. Input images were resized to 224 × 224 pixels, converted to grayscale, normalized by channel-wise statistics computed from the training set, and augmented using random transformations (cropping, rotations, horizontal flips, affine adjustments, perspective distortions, and brightness/contrast variations). The dataset was randomly divided into training (80%) and validation (20%) subsets, while preserving class distributions.

The models are trained for 100 epochs, using a batch size of 64 and the Adam optimizer. For loss we used cross-entropy with class weights proportional to the inverse frequency of each class, to address possible severe class imbalance in the data. We used dropout regularization (probability 0.3), early stopping (halting training if validation loss ceased improving), and adaptive learning rate scheduling (automatically reducing learning rate upon validation plateau) to ensure no overfitting.

For more detailed information, please refer to the documentation in our GitHub repository (https://github.com/biophysics-ul/rtdc-toolbox).

### Metabolic profiling using SeaHorse analyzer

Jurkat cells were seeded on 12-well cell culture plates at 2.5 × 10^5^ cells/mL and treated for 48 h with 0.1 mg/mL SMOF and 0.01 mg/mL Omegaven ILEs. After treatment, the cells were processed as described by Repas et al.^23^. Briefly, the cells were spun down and resuspended in Seahorse XF RPMI 1640-based Seahorse XF Glycolytic Rate Assay medium (2 mM glutamine, 1 mM HEPES, 0 mM pyruvate, 5.6 mM glucose) equilibrated to pH 7.4 and plated on Seahorse cell culture microplates covered with CellTak (Corning, Corning, NY, USA) at 125,000 cells in 0.1 mL per well. Plates were spun down at 200 g for 1 min and incubated at 37°C without CO_2_ for 15 min, after which 0.4 mL of the medium was added. After additional 30 min of incubation at 37°C without CO_2_, a Seahorse Mito Stress Assay modified to assess the contribution of beta oxidation was performed according to the manufacturer’s instructions using the Seahorse XFe24 analyzer (Agilent Technologies, Santa Clara, CA, USA). Following equilibration, three measurements were obtained, after which either medium or 5 µM etomoxir was injected, with the last of the five following measurements designated as the baseline oxygen consumption rate (OCR) and extracellular acidification rate (ECAR). After this, 1.5 μM oligomycin, 1.5 μM FCCP and 0.5 μM rotenone/antimycin A were sequentially injected to measure ATP-synthase independent OCR, maximal OCR and non-mitochondrial OCR, respectively.

The ATP production rate from oxidative phosphorylation (OxPhosATP) was calculated as previously described by Repas et al.^23^.

### Statistical analysis

The statistical analysis was performed using GraphPad Prism (v10.5; GraphPad Software, San Diego, CA, USA).

Data are presented as the mean ± SEM. One-way ANOVA was used for experiments where the data from each experiment was normalized to the respective control. Repeated measures one-way ANOVA was applied to all other data where matched data were available. In cases where values were missing within repeated measures datasets, the program automatically applied mixed-effects analysis. For experiments involving two independent variables, a two-way ANOVA was used. Post hoc tests were selected based on the experimental design: Dunnett’s test was applied when comparing all groups to a control, and Tukey’s test was used for multiple comparisons between all groups. A *p*-value of < 0.05 was considered statistically significant.

## Results

### Automated classification of deformability cytometry events based on supervised machine learning

DC images of Jurkat cells incubated in LEs revealed that many of the detected events did not represent intact cells. In cytometry, these unwanted events are typically excluded through manual gating. In DC, for example, objects below a certain size are often classified as debris and excluded based solely on size^19^. However, manual gating is labor-intensive and subject to operator variability. On the other hand, DC generates clear images of all events, allowing different event types to be visually distinguished. Building on this capability, we developed a deep neural network for automatic, gate-free classification of events observed in the data.

**Fig. 2:**
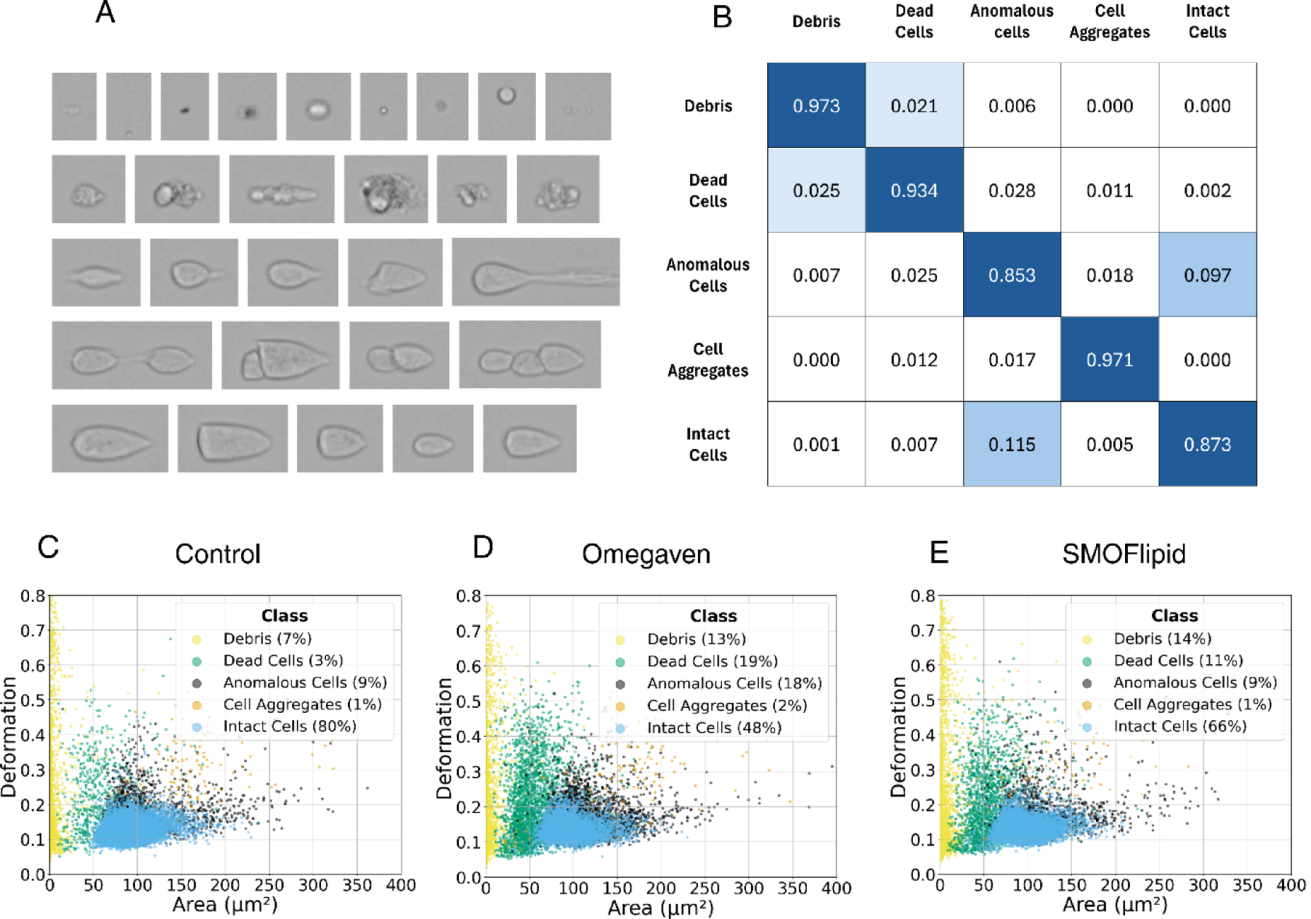
Machine-learning-based classification of images. (**A**) Examples of distinct image classes observed in samples of Jurkat cells (from top to bottom): debris, dead cells, anomalous cells, cell aggregates, and intact cells. Note that the distinction between intact and anomalous cells is not always clear, even to the human eye. Additionally, some cell aggregates appear smaller than intact cells. (**B**) Confusion matrix, illustrating the performance of the supervised machine-learning classification model based on a deep neural network. (**C**)–(**E**) Typical examples of a deformation–area scatter plots of automatically classified cells: (**C**) control cells (N = 21,391), (**D**) cells incubated in Omegaven (0.01 mg/mL, N = 12,020) and (**E**) cells incubated with SMOFlipid (0.1 mg/mL; N = 15,841). These concentrations were the highest at which cell viability after 48 h of incubation remained sufficient for further analysis with deformability cytometry. The fraction of events classified as dead cells or debris is clearly higher in the Omegaven- and SMOFlipid-treated samples compared to the control.

Based on a manual inspection of the images, we identified five distinct image classes: debris, dead cells, cell aggregates, anomalous cells, and intact cells (Fig. 2A). Note that some anomalous cells are larger than intact cells, while some aggregates are smaller, highlighting the limitations of size-based gating. We therefore manually prepared a training set consisting of 4,904 intact cells, 1,456 anomalous cells, 2,525 dead cells, 784 cell aggregates, and 1,829 debris images. While preparing the training set, we found that distinguishing between intact and anomalous cells in borderline cases was often challenging, even for human observers. To ensure consistency, the dataset was therefore reviewed by two independent operators.

Model training took approximately one hour on a personal computer equipped with a mid-range GPU (see Supplementary Figure S3). As shown in the confusion matrix (Fig. 2B), the trained model demonstrated excellent accuracy in identifying debris and cell aggregates (97%). The accuracy of detecting dead cells was slightly lower (93%), while the lowest accuracies were observed for intact and anomalous cells (87% and 85%, respectively). This result was expected, as these classes had already proven to be the most difficult to distinguish during training set preparation.

Applying the classification model to a control sample revealed substantial overlap between the different cell classes in the standard DC scatter plot of deformation versus area (Fig. 2C). This overlap became even more pronounced in cells exposed to an ILE (Figs. 2D, E). We therefore omitted manual gating and relied solely on the deep neural network model to classify all our DC data.

### Intravenous lipid emulsions are toxic to Jurkat cells, which can be detected ‘label-free’ by deformability cytometry image classification

**Fig. 3:**
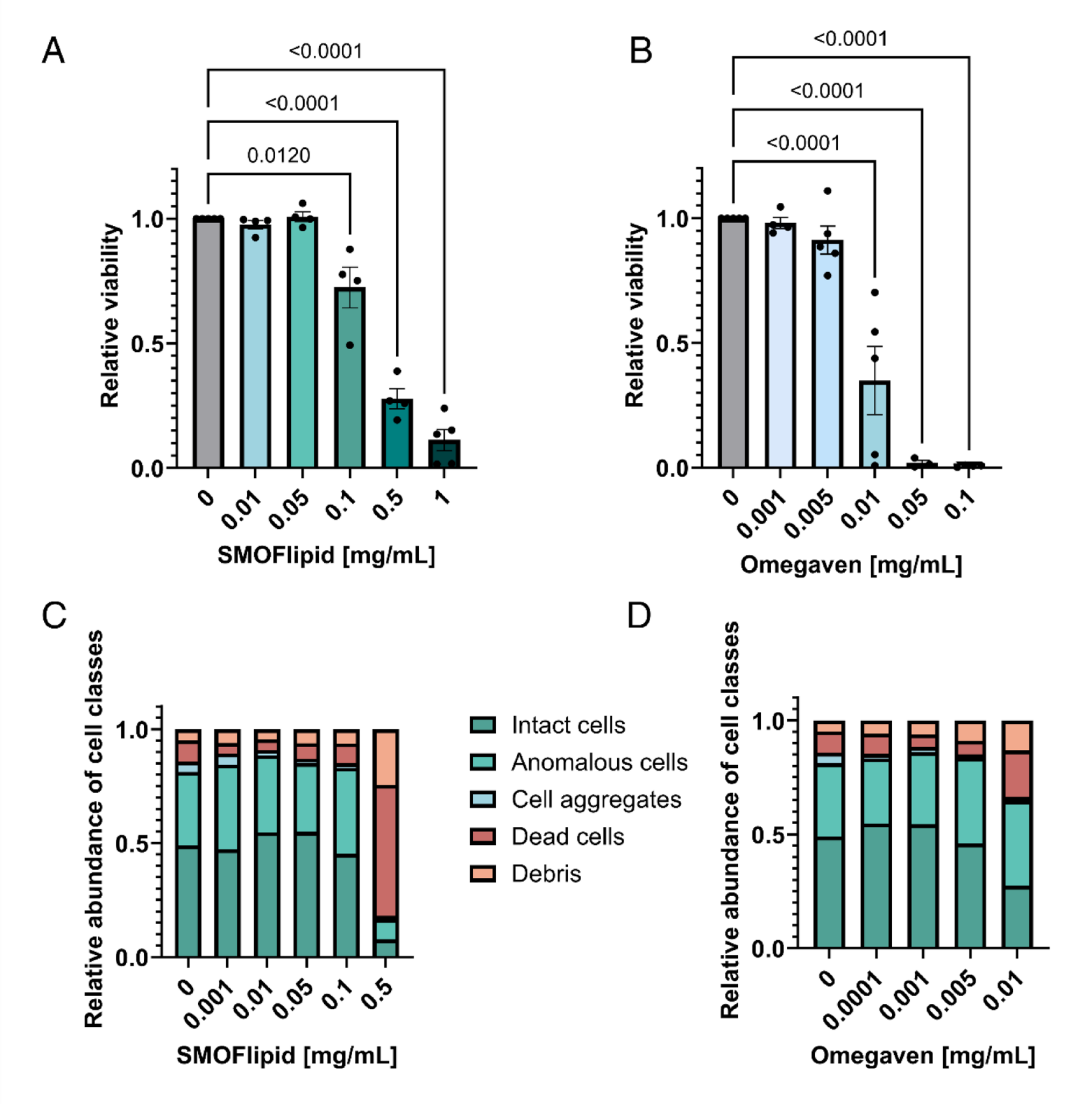
Effect of lipid emulsions on the viability of non-activated Jurkat cells. The cells were incubated for 48 h in SMOFlipid (**A**, **C**) or Omegaven (**B**, **D**). (**A**) and (**B**): relative viability measured by MTS assay, calculated as the absorbance at 490 nm relative to the control. Each data point represents the average of 4 to 5 technical replicates. Bar plots show the mean ± SEM, and bar colors indicate lipid emulsion concentrations. Statistical significance was assessed using one-way ANOVA followed by Dunnett’s post hoc test, comparing all samples to the control. (**C**) and (**D**): relative abundance of five image classes—intact cells, anomalous cells, cell aggregates, dead cells, and debris—based on image-based classification from deformability cytometry, illustrating the overall cell state after incubation. Results represent the average cell class abundances from 5 to 6 replicates, each containing at least 3,000 events.

As high lipid concentrations are known to be toxic to cells and may contribute to the adverse side effects associated with the clinical use of lipid emulsions, we first evaluated the effects of the lipid emulsions SMOFlipid and Omegaven on the viability of Jurkat cells. Using the standard MTS viability assay, we tested a range of lipid concentrations up to the expected physiological levels during emulsion therapy, which is approximately 0.4 mg/mL for SMOFlipid and 0.1 mg/mL for Omegaven (see Supplementary tables S1, S2, S3). We found that SMOFlipid started to exhibit toxicity at a lipid concentration of 0.1 mg/mL (Fig. 3A), while Omegaven showed toxicity at lipid concentrations as low as 0.01 mg/mL (Fig. 3B). This result indicates that Omegaven is approximately ten times more toxic to Jurkat cells than SMOFlipid. We also tested the effects of pure FAs on the viability of our Jurkat cells (see Supplementary figure S4), which corroborated findings from the literature^8,9,24^. The cells were significantly more susceptible to omega-3 FAs (EPA and DHA) than to OA and LA.

As automated classification in DC provided the percentage of specific cell classes in our samples and detected shifts in cell populations upon incubation with lipid emulsions (Fig. 2C–E), we next explored the potential of DC as a label-free method for assessing cell viability. Figures 3C, D show that changes in cell class fractions with increasing lipid concentrations correlate well with the results of the MTS assay, indicating that DC can indeed be used as a simple and rapid label-free method to assess cell state.

### Deformability cytometry reveals limited mechanical alterations in Jurkat cells treated with lipid emulsions

**Fig. 4:**
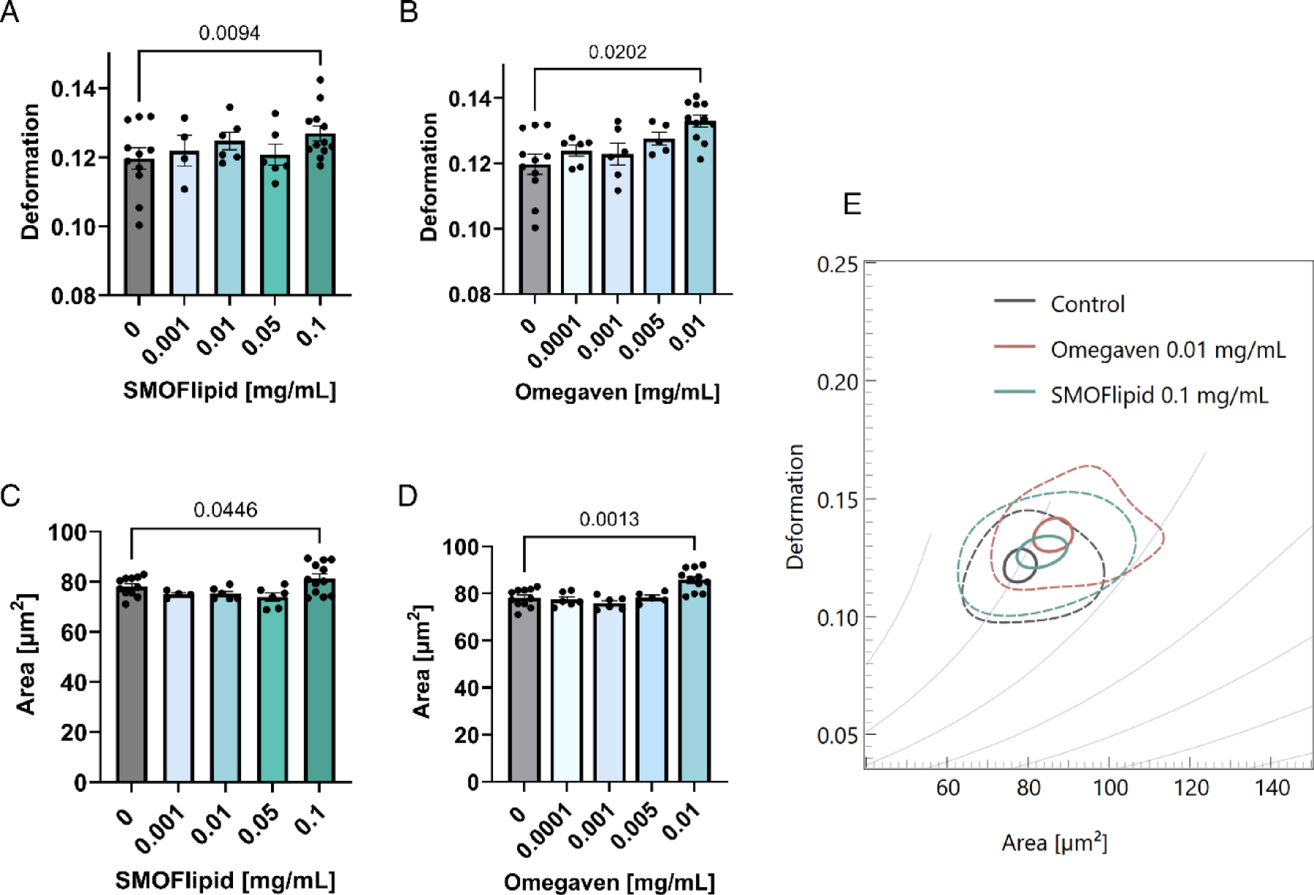
Effect of lipid emulsions on the deformation and size (projected area) of non-activated Jurkat cells. The cells were incubated for 48 h in SMOFlipid (**A**, **C**) or Omegaven (**B**, **D**). Each data point on the graph represents the mode of measurements of at least 900 cells analyzed from a single experiment. Bar plots show the mean ± SEM of these values, and bar colors indicate lipid emulsion concentrations. Statistical significance was assessed using mixed effects analysis followed by Dunnett’s post hoc test, comparing all samples to the control. (**E**) A typical example of density plots showing differences in cell size (projected area) and deformation for control cells and cells treated for 48 h with 0.01 mg/mL Omegaven or 0.1 mg/mL SMOFlipid. The grey lines represent theoretical isoelasticity curves calculated by CytoPlot analysis (calculation model: LE-2D-FEM-19).

To investigate whether lipid emulsions alter the mechanical properties of Jurkat cells, we performed deformability cytometry following incubation with the same concentrations of SMOFlipid and Omegaven as used in the viability assays (SMOFlipid ranged from 0.01 mg/mL to 1 mg/mL, and Omegaven from 0.001 mg/mL to 0.1 mg/mL). We observed a slight increase in cell deformation after lipid treatment; however, this effect only reached statistical significance at the highest tested concentrations for both emulsions (Figs. 4A, B). In parallel, we noted a slight increase in the projected cell area at these same concentrations (Figs. 4C, D). These results suggest that although lipid emulsions can mildly change the measured deformability of Jurkat cells, the overall intrinsic stiffness of cells was not substantially affected as these changes are along the theoretical iso-elasticity lines (Fig. 4E).

### Lipid droplet accumulation in Jurkat cells increases with lipid emulsion concentration, which can be detected by deformability cytometry image analysis

**Fig. 5:**
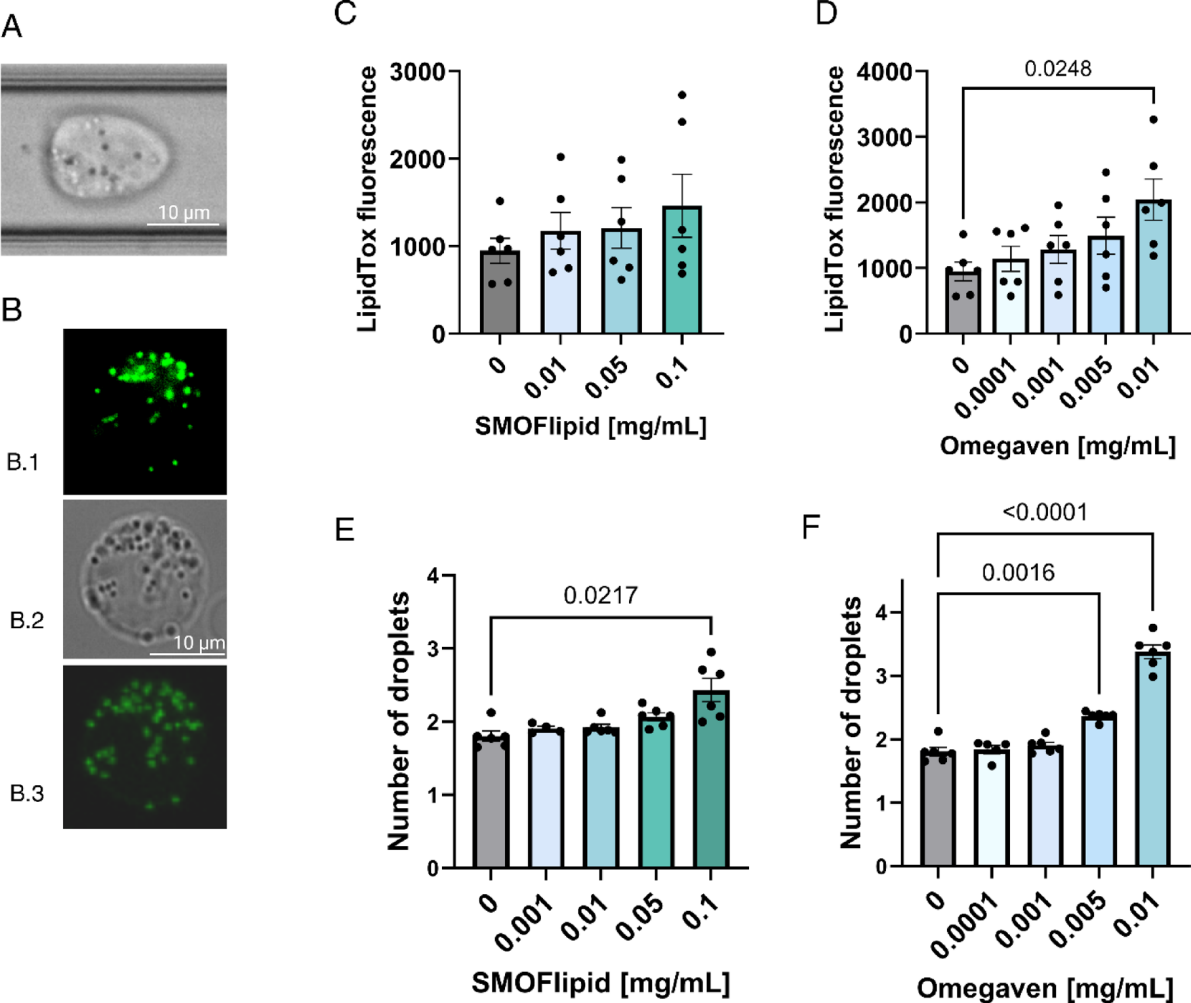
Lipid droplet accumulation in non-activated Jurkat cells incubated for 48h with lipid emulsions. (**A**) A brightfield image from deformability cytometry highlighting a Jurkat cell with visible lipid droplets. (**B**) Representative brightfield and LipidTOX-stained fluorescence microscopy images of Jurkat cells after incubation with lipid emulsions, showing intracellular lipid droplets B.1—epifluorescence, B.2—brightfield and B.3—maximum intensity projection confocal microscopy image. (**C**) and (**D**): flow cytometry analysis showing mean LipidTOX fluorescence intensity of single cells positive for LipidTOX in response to increasing concentrations of lipid emulsions SMOFlipid (**C**) and Omegaven (**D**). Each data point on the graph represents the average measurement of 30,000 cells. Bar plots show the mean ± SEM of these values, and bar colors indicate lipid emulsion concentrations. Statistical significance was assessed using repeated measures one-way ANOVA followed by Dunnett’s post hoc test, comparing all samples to the control. (**E**) and (**F**): label-free quantification of average number of lipid droplets per cell using deformability cytometry across different concentrations of SMOFlipid (**E**) and Omegaven (**F**). Each data point on the graph represents the average number of lipid droplets measured in at least 900 cells from a single experiment. While the number of lipid droplets per cell is always an integer, the displayed values are averages across many cells and are therefore expressed as non-integer numbers. Bar plots show the mean ± SEM of these values, and bar colors indicate lipid emulsion concentrations. Statistical significance was assessed using mixed effects analysis followed by Dunnett’s post hoc test, comparing all samples to the control.

Manual inspection of deformability cytometry images revealed the presence of dark inclusions within the cytoplasm of cells incubated in lipid emulsions (Fig. 5A). To investigate further, we stained the cells with fluorescent lipid marker LipidTOX and inspected them under confocal microscopy, which confirmed that these structures were intracellular lipid droplets (Fig. 5B). To quantify this observation, we analyzed LipidTOX stained cells by flow cytometry, which showed a consistent trend of increasing fluorescence intensity with higher emulsion concentrations (Figs. 5C, D).

Next, we developed a simple thresholding algorithm to quantify the number of inclusions in the DC images in a label-free manner (see Supplementary Figure S5). This analysis revealed a clear concentration-dependent increase in the accumulation of lipid droplets in cells treated with both SMOFlipid and Omegaven (Figs. 5E, 5F). Notably, Omegaven induced significantly more lipid droplet formation than SMOFlipid, achieving this at lower concentrations. This mode of quantification is one possible approach, but not the only one. For an alternative method using the average lipid droplet area per cell, see Supplementary Figure S6.

Taken together, these results suggest that exposure of Jurkat cells to lipid emulsions leads to lipid uptake, droplet formation and intracellular accumulation, with the latter occurring more prominently in response to the fish oil-based formulation Omegaven. In addition, DC enabled both label-free visualization and quantification.

### Lipids from lipid emulsions do not alter the energy metabolism of non-activated Jurkat cells

**Fig. 6:**
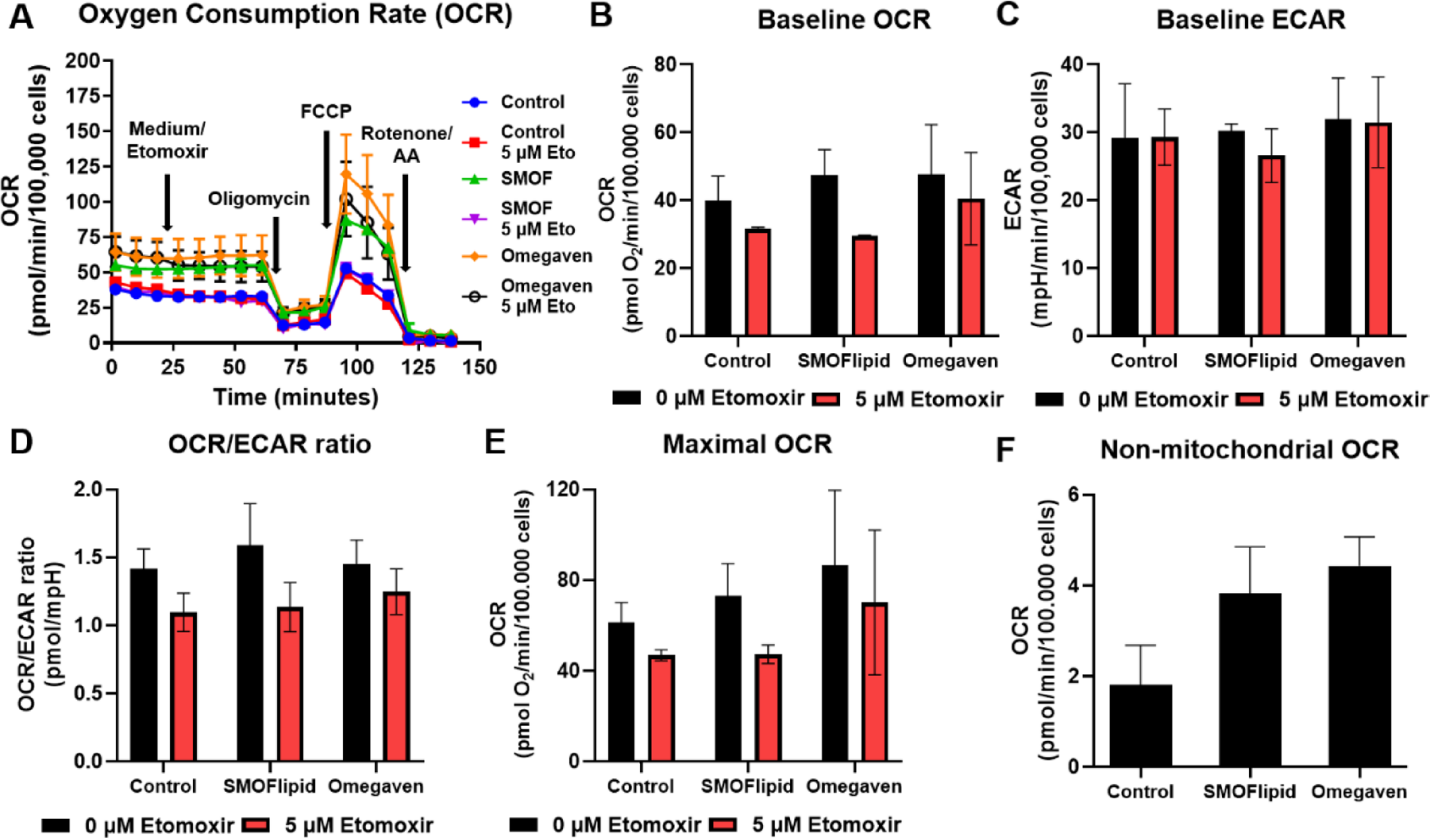
The effect of lipid emulsions on energy metabolism of non-activated Jurkat cells. The cells were incubated for 48h at 0.1 mg/mL lipid concentration for SMOFlipid and 0.01 mg/mL for Omegaven, after which the rate of oxygen consumption (OCR) and extracellular acidification rate (ECAR) were determined using a modified Seahorse Mito Stress Assay with a prior etomoxir injection. The assay is used to measure the contribution of beta oxidation, the primary metabolic pathway for fatty acid catabolism. No significant altered baseline or maximal oxygen consumption rate (OCR), baseline extracellular acidification rate (ECAR) or the ratio between the two (OCR/ECAR) were observed (B–E). (**A**) A representative OCR graph for one experiment. After 3 measurement cycles, 5 µM etomoxir or medium was injected, after which baseline OCR (**B**) and ECAR (**C**) were measured and the OCR to ECAR ratio (**D**) was calculated. 1.5 µM oligomycin, 1.5 µM carbonyl cyanide p-trifluoromethoxyphenylhydrazone (FCCP), and 0.5 µM rotenone + antimycin A (AA) were then sequentially injected to determine the ATP-synthase independent, maximal (**E**) and non-mitochondrial (**F**) OCR, respectively. Data represent mean ± SEM of two independent experiments. No statistically significant differences were found by two-way (**B**–**E**) or one-way (F) ANOVA with Dunnett’s post-hoc test.

To investigate whether fatty acids from lipid emulsions in extracellular space or lipid droplets in the cytoplasm are used for energy metabolism of Jurkat cells we next investigated the effect of lipid emulsions on Jurkat cell metabolism using the Seahorse Mito Stress assay modified to estimate the contribution of beta oxidation to oxygen consumption, which represents the primary metabolic pathway for lipid catabolism (Fig. 6A). We found that neither SMOFlipid nor Omegaven significantly altered baseline or maximal oxygen consumption rate (OCR), baseline extracellular acidification rate (ECAR) or the ratio between the two (OCR/ECAR) (Figs. 6B–E). The same trend was observed for ATP production from oxidative phosphorylation and glycolysis, as well as their ratio (see Supplementary Figure S7). The injection of beta oxidation inhibitor etomoxir did slightly but consistently decrease both baseline and maximal OCR, as well as the OCR/ECAR ratio (not statistically significant) in control cells, indicating a small but noticeable contribution of fatty acid oxidation to total oxidative metabolism. This contribution was essentially unchanged by SMOFlipid treatment and possibly even slightly reduced by Omegaven, suggesting lipid emulsions did not increase mitochondrial fatty acid beta oxidation. There was also no major increase in non-mitochondrial OCR (Fig. 6F) compared to control to suggest increased peroxisomal fatty acid beta oxidation with either emulsion. Overall, the lipid emulsion treatment did not significantly alter Jurkat cell energy metabolism, and, importantly, did not substantially increase the rate of fatty acid beta oxidation either inside or outside mitochondria.

### Activated Jurkat cells show increased sensitivity to lipid emulsions without increase in accumulation of lipid droplets

**Fig. 7:**
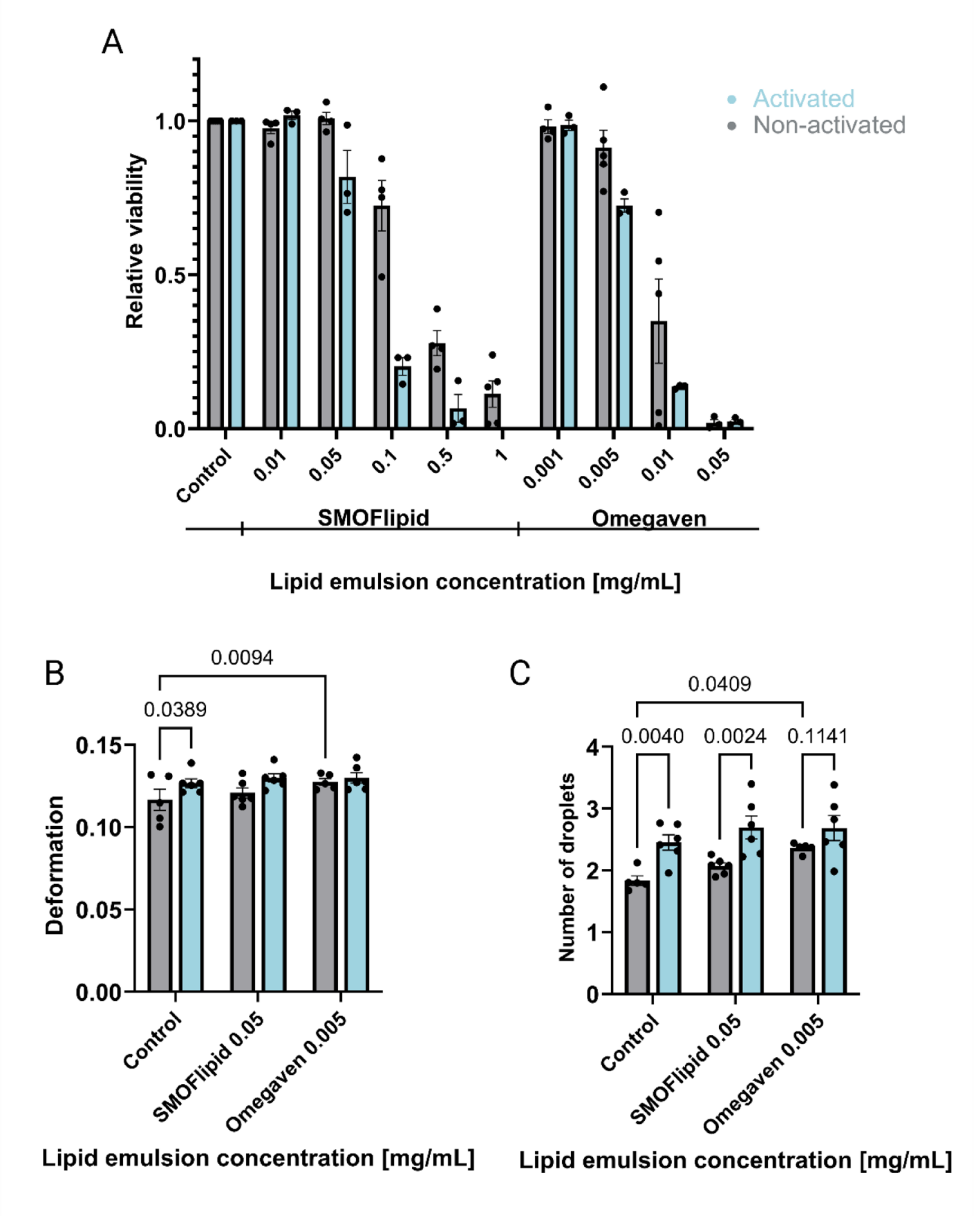
Impact of lipid emulsions on activated versus non-activated Jurkat cells. The cells were incubated for 48 h in lipid emulsions SMOFlipid and Omegaven. (**A**) Cell viability was assessed using an MTS assay with increasing concentrations of SMOFlipid and Omegaven for both non-activated and activated Jurkat cells. Relative viability was calculated by comparing the absorbance value at 490 nm for incubated cells with that for their respective controls. Each data point on the graph represents an average of 4 technical replicates. Bar plots show the mean ± SEM of these values. (**B**) Comparison of cell deformability of non-activated and activated Jurkat cells measured by deformability cytometry. Statistical significance was assessed using mixed effects analysis followed by Šidak’s multiple comparison test. (**C**) Quantification of lipid droplet formation using deformability cytometry image analysis. Statistical significance was assessed using two-way ANOVA with multiple comparisons. Each data point on the graph represents the mode (**B**) or mean (**C**) of at least 1,000 measured cells in one experiment. Bar plots show the mean ± SEM of these values.

To investigate how activation influences the response of Jurkat cells to intravenous lipid emulsions, the cells were first activated and then incubated for 48 h with varying concentrations of SMOFlipid or Omegaven, using the same protocols as for non-activated cells. Cell viability, assessed via the MTS assay, showed a concentration-dependent decrease for both lipid emulsions (Fig. 7A). Compared to non-activated cells, activated Jurkat cells exhibited approximately twice the sensitivity to both emulsions (Fig. 7A), indicating enhanced vulnerability upon activation. Compared to non-activated cells, DC revealed even less changes in the mechanical properties of activated Jurkat cells following lipid treatment (Fig. 7B). Additionally, label-free, image-based analysis of intracellular lipid droplets revealed a higher presence of lipid droplets even prior to incubation with lipid emulsions, with no detectable accumulation observed in activated cells following exposure to either SMOFlipid or Omegaven (Fig. 7C). This absence of droplet formation suggests that activation may alter lipid handling pathways or cellular uptake mechanisms.

## Discussion

The immunomodulatory effects of fatty acids are well established but not yet fully understood. In this work, we presented a comparative study of the effects of two widely used intravenous lipid emulsions (ILEs) on Jurkat cells. Our aim was to contribute to the understanding of how these emulsions may influence lymphocyte fitness, which is a topic of growing importance in the context of immunotherapy. This is particularly relevant since patients receiving such therapies often also require parenteral nutrition with ILEs. We employed deformability cytometry (DC) as our primary analytical tool. This novel, label-free technique is simple to operate and requires only a small sample of cells, making it a promising candidate for monitoring the status of blood cells in critically ill patients.

The study focused on SMOFlipid and Omegaven, which are the most used formulations in clinical nutritional support for patients requiring parenteral nutrition. Their markedly different fatty acid compositions (see Supplementary Tables S1, S2) may have distinct effects on immune cells, but these have not been systematically examined. Omegaven is rich in omega-3 PUFAs, mainly EPA and DHA, and together constitute 40–54% of its total fatty acid content. In contrast, SMOFlipid is a new-generation, more balanced emulsion that contains a mixture of soybean oil, medium-chain triglycerides, olive oil, and fish oil, resulting in higher levels of OA and LA. These differences in composition are critical, as omega-3 PUFAs are known to exert both cytotoxic and immunomodulatory effects on immune cells^25–28^, including modulation of receptor signaling^29,30^ and mechanical properties^31^. In contrast, OA and LA are generally considered to be less immunoactive, and the literature on their effects is more limited and inconclusive.

In our study, we observed a concentration-dependent decrease in the viability of Jurkat cells incubated with both SMOFlipid and Omegaven ILE (Fig. 3). The highest concentrations used were within the clinically relevant range, as estimated from pharmacokinetic calculations (see Supplementary Table S3) and supported by measurements from blood samples of patients receiving lipid emulsions as nutritional support^32^. Consistent with previously reported toxic effects of FAs on Jurkat cells and T lymphocytes, we found that Omegaven induced toxicity at concentrations approximately ten times lower than those required for SMOFlipid (Fig. 3). To our knowledge, this is the first comparative in vitro study to examine the effects of clinically used ILEs on T cell viability using the Jurkat cell model.

Earlier comparative studies have demonstrated that acute myeloid leukemia (AML) cell lines are more susceptible to viability loss when treated with EPA and DHA (both omega-3 polyunsaturated fatty acids–PUFAs) than with OA (omega-9)^33^. Studies conducted on Jurkat cells have found that OA is less toxic than LA (omega-6)^8^ and that DHA inhibits cell proliferation more strongly than EPA^24^. We corroborated these findings through our screening of the toxicity of free EPA, DHA, OA, and LA (see Supplementary Figure S4). It is important to note that the concentrations of free FAs cannot be directly compared to the lipid concentrations in ILEs, as FAs in ILEs are stored in triglyceride form within lipid droplets. Still, the results with free FAs indicate that the observed differences in toxicity of ILEs are likely to be rooted in the different biological impact of these FAs and not just in the total lipid content. This underscores the need for a more precise evaluation of the ILE effects in clinical settings, particularly in view of the growing use of immunotherapies such as CAR-T therapy in clinical practice. Although our results were obtained using Jurkat cells (a leukemia-derived T-cell line), they highlight important mechanisms that merit further investigation in primary immune cells.

A key innovation of this study was the application of DC to investigate the mechanical properties and morphological features of individual cells. We hypothesized that mechanical properties such as cell deformability might be altered by exposure to Omegaven, as PUFAs such as DHA are highly flexible and are known to increase membrane fluidity and flexibility^30,34–37^. Notably, EPA and DHA have been shown to increase membrane fluidity significantly more than LA in CD4⁺ T cells^36^. In addition, an in vivo study reported increased lymphocyte deformability in participants after 12 weeks of fish oil supplementation^31^.

However, we observed only a slight effect on the deformability of Jurkat cells (Fig. 4), suggesting that changes in membrane composition alone may not be sufficient to significantly alter cell mechanics. This points to important role of the cytoskeleton and the nucleus in determining Jurkat cell stiffness and deformability. There is currently little known about how lipid emulsions influence the cytoskeletal architecture of immune cells, making this an important avenue for future research.

Our study expanded the conventional use of DC as a label-free tool in two novel directions. First, we developed a supervised machine learning–based classification model to automatically distinguish between different classes of cell images, such as intact cells, dead cells, cell aggregates and debris (Fig. 2). While other models for DC image classification have been described in the literature^38^, our goal was to develop a robust model that could handle images of varying sizes and require no detailed knowledge of AI for its operation. This enabled automated analysis of DC data without the need for manual gating. Such automatic gating is also becoming a standard approach in conventional flow cytometry^39^. Compared with prior machine-learning classification in DC using advanced image-based algorithms^38^, full sequences of single-cell images^40,41^, or scalar features from segmented cells^42^, our goal was to develop a robust model that requires no specialized AI expertise to operate and does not rely on the specifics of image segmentation and feature extraction. Using this automated analysis, we were able to identify changes in cell state in a high-throughput, label-free manner and detect reductions in viability that were consistent with those observed using the standard MTS assay (Fig. 3). These findings demonstrate the potential of DC as a non-invasive tool for assessing cell fitness and highlight a novel application of the technique.

The second important observation in our DC study was the accumulation of intracellular lipid droplets. Cytoplasmic inclusions were first noted in DC, prompting us to extend the standard DC analysis by quantifying these inclusions. A clear increase in their number was observed with rising ILE concentrations (Fig. 5). Subsequent staining with LipidTOX and inspection with fluorescence microscopy and flow cytometry confirmed that the inclusions were lipid droplets. Notably, lipid droplet accumulation was much more pronounced in cells treated with Omegaven than with SMOFlipid, correlating with the observed cytotoxicity.

Motivated by reports showing that lipids can impair immune cell metabolism, promote exhaustion, reduce cell fitness, and ultimately weaken the anti-tumor immune response^43–45^, we analyzed the metabolism of Jurkat cells using a Seahorse analyzer (Fig. 6). However, treatment with ILEs did not result in significant metabolic alterations: mitochondrial respiration and glycolysis remained largely unaffected, and overall ATP production was unchanged. These results suggest that the increased internalization of FAs does not enhance their utilization in energy metabolism. Instead, our data indicates a preferential sequestration of excess lipids into intracellular lipid droplets. This is consistent with previous findings that one of the key cellular defense mechanisms against lipid overload is the formation of lipid droplets, which sequester lipids intracellularly and help mitigate lipotoxicity^46,47^. Lipid droplet formation in T cells has also been described as a response to high-lipid environments^48,49^.

Additionally, we tested how activation of Jurkat cells affects their response to ILEs and found that activated Jurkat cells exhibited higher baseline levels of lipid droplets compared to non-activated cells but did not form additional droplets upon exposure to lipid emulsions (Fig. 7). This suggests that activated cells may have altered lipid storage mechanisms, which could contribute to the increased susceptibility to lipotoxicity observed in these cells (Fig. 7). Importantly, our results indicate that Jurkat cells are not an ideal model for studying cellular mechanical properties, as they do not accurately replicate the behavior of primary T cells in response to activation. Unlike primary lymphocytes, which typically exhibit substantial changes in size, morphology, and mechanical properties upon activation^50^, activated Jurkat cells in our experiments showed only minimal changes in size and deformability (Fig. 7, Supplementary Figure S8). Therefore, the observed small effects of ILEs on the deformability of Jurkat cells may not accurately reflect the response of primary lymphocytes.

In conclusion, we employed deformability cytometry (DC) to assess the effects of two intravenous lipid emulsions (ILEs) on Jurkat cells. While our study did not reveal major changes in cell mechanics, it showed that the fish oil–based ILE Omegaven induced greater cytotoxicity than the more balanced formulation SMOFlipid. This cytotoxic effect correlated with the accumulation of intracellular lipid droplets. As confirmed by using a Seahorse analyzer, neither ILE caused significant alterations in cellular metabolism. Furthermore, we demonstrated the potential of DC as a simple, non-invasive, and label-free tool for assessing cell state. We have made all DC images and the analysis code publicly available in an open repository. We believe that this resource will contribute to the development and validation of DC as a robust, transparent, and reproducible analytical tool. Together, our findings provide a solid foundation for future investigations on primary lymphocytes, which will be crucial for advancing our understanding of the immunotoxicological impact of lipid emulsions and ensuring their safe use in patients undergoing advanced immunotherapies.

## Supplementary Information

Below is the link to the electronic supplementary material.

Supplementary Material 1

## Data Availability

Data that support the findings of this study are available from the corresponding author upon reasonable request. The raw deformability cytometry images have been deposited to [https://cloud.elixir-hpc.si/DeformabilityCytometry] (https:/cloud.elixir-hpc.si/DeformabilityCytometry).
