# Supplementary material for: Effects of intravenous lipid emulsions on Jurkat cells assessed using label-free deformability cytometry
Source: Sci Rep. 2025 Dec 30;16:3123. doi: 10.1038/s41598-025-33582-7 (PMC12830582; doi:10.1038/s41598-025-33582-7)
Supplement: Supplementary file 1 — Supplementary Material 1 [file 41598_2025_33582_MOESM1_ESM.docx]

SUPPLEMENTAL INFORMATION

**Effects of intravenous lipid emulsions on Jurkat cells assessed using label-free deformability cytometry**

Lija Fajdiga^1^, Jernej Repas^1^, Bor Ivanuš^2^, Darin Lah^1^, Nina Bernat^1^, Lara Betocchi^1^, Miran Bürmen^2^, Špela Zemljič^1^ in Jure Derganc^1*^

^1^Institute of Biophysics, Faculty of Medicine, University of Ljubljana, Ljubljana, Slovenia

^2^Faculty of Electrical Engineering, University of Ljubljana, Ljubljana, Slovenia

* Corresponding author


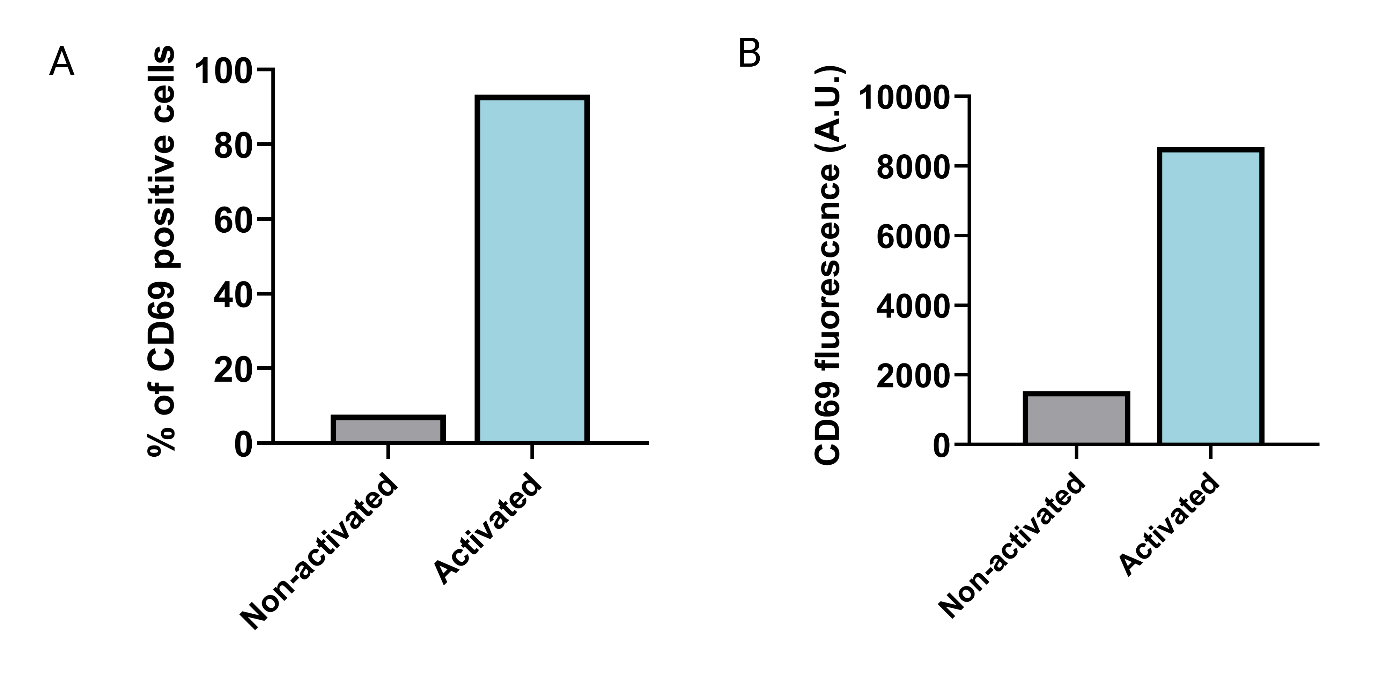


**Supplemental Figure S1: Comparison of CD69 expression between non-activated and activated Jurkat cells**. Jurkat cells were activated for 48 hours using anti-CD3/CD28 antibodies. CD69 surface expression was assessed by flow cytometry using fluorescently labeled anti-CD69 antibodies. The bar plots show (A) the percentage of CD69-positive cells and (B) the mean fluorescence intensity (MFI) of CD69 expression in non-activated and activated cells. With this test we confirmed successful activation of Jurkat cells.

| 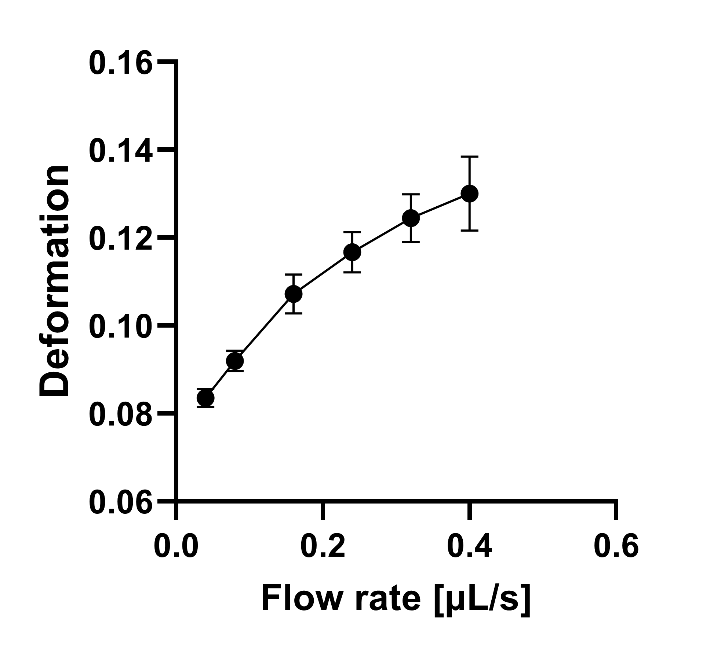 |
| --- |
| **Supplemental Figure S2: Validation of custom-made deformability cytometry system with Jurkat cells.** The custom-built DC system was validated by increasing the flow rate, which caused an increase in cell deformation as expected. Each data point on the graph represents the average of 4 independent replicates (with each experiment comprising measurements from at least 5,500 cells) ± SEM. |

| A)  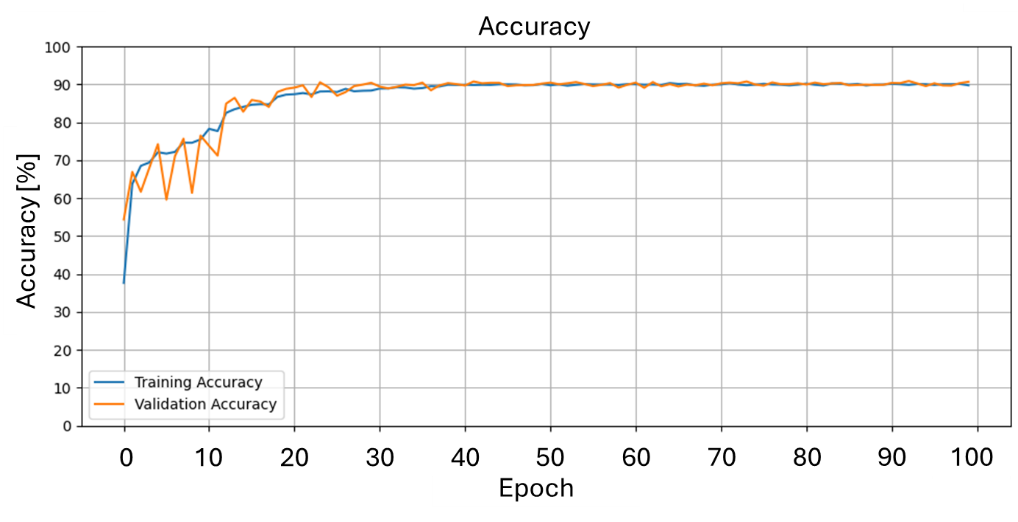  B)  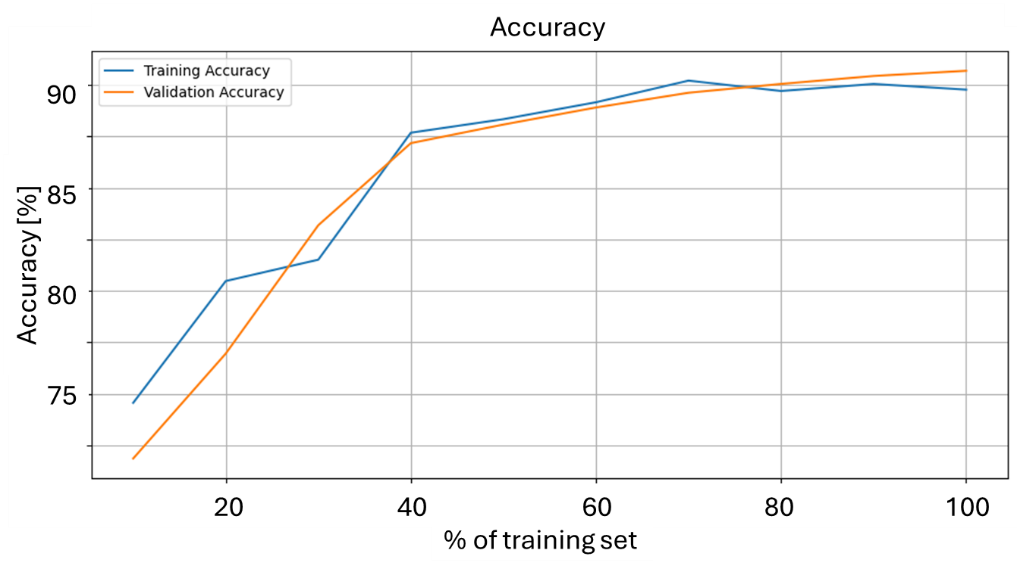 |
| --- |
| **Supplement Figure S3. Efficiency of the CNN model training.** A) Model accuracy over training epochs. B) Effect of training set size on the model accuracy. The training on the whole training set took approximately 1 h for 100 epochs on NVIDIA GPU RTX 3060 (~ 400 € at the time of purchase). |

**Supplemental Table S1: Composition of Omegaven lipid emulsion as provided by the manufacturer**

| COMPONENT | Content per 1 mL of Omegaven | |
| --- | --- | --- |
| Fish oil | 0.1 g | EPA (13-26 %),  DHA (14% to 27%),  palmitic acid (4% to 12%),  oleic acid (4% to 11%),  palmitoleic acid (4% to 10%),  myristic acid (2% to 7%),  arachidonic acid (0.2% to 2.0%),  linoleic acid (1.5%),  alpha linolenic acid (1.1%). |
| Egg phospholipids | 0.012g | |
| Gycerin | 0.025 g | |
| Alpha-tocopherol | 0.15 – 0.3 mg | |
| Sodium oleate | 0.3 mg | |
| Water for injection |  | |
| NaOH (for pH adjustment) |  | |

**Supplemental Table S2: Compositions of SMOFlipid emulsion as provided by the manufacturer**

| COMPONENT | Content per 1 mL of SMOFlipid | |
| --- | --- | --- |
| Soybean oil | 60 mg | Oleic acid (23 % to 35 %),  linoleic acid (14% to 25%),  caprylic acid (13% to 24%),  palmitic acid (7% to 12%),  capric acid (5% to 15%),  stearic acid (1.5% to 4%),  alpha-linolenic acid (1.5% to 3.5%),  EPA (1% to 3.5%),  DHA (1% to 3.5%). |
| MTC | 60 mg |  |
| Olive oil | 50 mg |  |
| Fish oil | 30 mg |  |
| Egg phospholipids | 0.012g | |
| Gycerin | 0.025 g | |
| Alpha-tocopherol | 0.163 – 0.225 mg | |
| Sodium oleate | 0.3 mg | |
| Water for injection |  | |
| NaOH (for pH adjustment) |  | |

**Supplementary Table S3: Pharmacological calculations of steady-state blood concentrations of Omegaven and SMOFlipid emulsions**

Here, we estimate the steady-state concentration of lipids in the blood during intravenous (IV) therapy with ILEs. The estimation is based on the recommended infusion rates of ILEs and literature-derived half-life values. Due to the lack of precise data on lipid elimination rates, we assumed first-order kinetics for lipid elimination in our calculations.

| Quantities used: | $V_{d}$: volume of distribution.  $T_{½}$: half-life for emulsions in blood.  $k_{el}$: elimination rate, given by the relation $k_{el}=\frac{\ln2}{T_{½}}$.  $k_{0}$: infusion rate.  $c_{SS}$: final (steady-state) concentration, given by the relation $c_{SS}=\frac{k_{0}}{k_{el}V_{d}}$. |
| --- | --- |

The data on the recommended doses and estimates of the elimination rates were obtained in the publicly available repository Medsafe, New Zealand Medicines and Medical Devices Safety Authority (<https://www.medsafe.govt.nz/index.asp>). Assuming a 70 kg person, the doses are:

| **Omegaven**  *Elimination data:*  $T_{½}$= 54 min = 0.9 h  $k_{el}=0.77/$h  *Daily dose of lipids:*  0.1g – 0.2g/kg  *Average infusion rate*  for a 70 kg person, assuming daily dose 0.15 g/kg administered over 24 h ⇒ $k_{0}=$0.44 g/h | **SMOFlipid**  *Elimination data:*  $T_{½}$ = 0.35 h  $k_{el}=1.98/$h  *Daily dose of lipids:*  1g - 2g/kg  *Average infusion rate*  for a 70 kg person, assuming daily dose 1.5 g/kg administered over 24 h ⇒ $k_{0}=$4.4 g/h |
| --- | --- |

Based on data from the literature, approximately 80% of the emulsion passes from the blood into other tissues as well (Hultin et al. 1995). In principle, the concentration of the emulsion should be calculated using a two-compartment model. However, due to insufficient data on the transfer rates between plasma and tissues, we used a one-compartment model, assuming that only 80% of the administered substance remains in the blood. Consequently, we approximate that the volume of distribution is greater than the plasma volume by a factor of 1/0.8.

An average person has 70 mL of blood / kg, hence the volume of distribution for a 70 kg person is

$V_{d}=\frac{70\text{ kg} \times70 \text{mL}}{\text{kg}}\times\frac{1}{0.8} \sim6000 \text{mL}$.

Assuming the average infusion rates the average steady-state blood concentrations as:

| **Omegaven**  $c_{SS} \sim0.1 \text{mg/mL}$ | **SMOFlipid**  $c_{SS} \sim0.4 \text{mg/mL}$ |
| --- | --- |

| 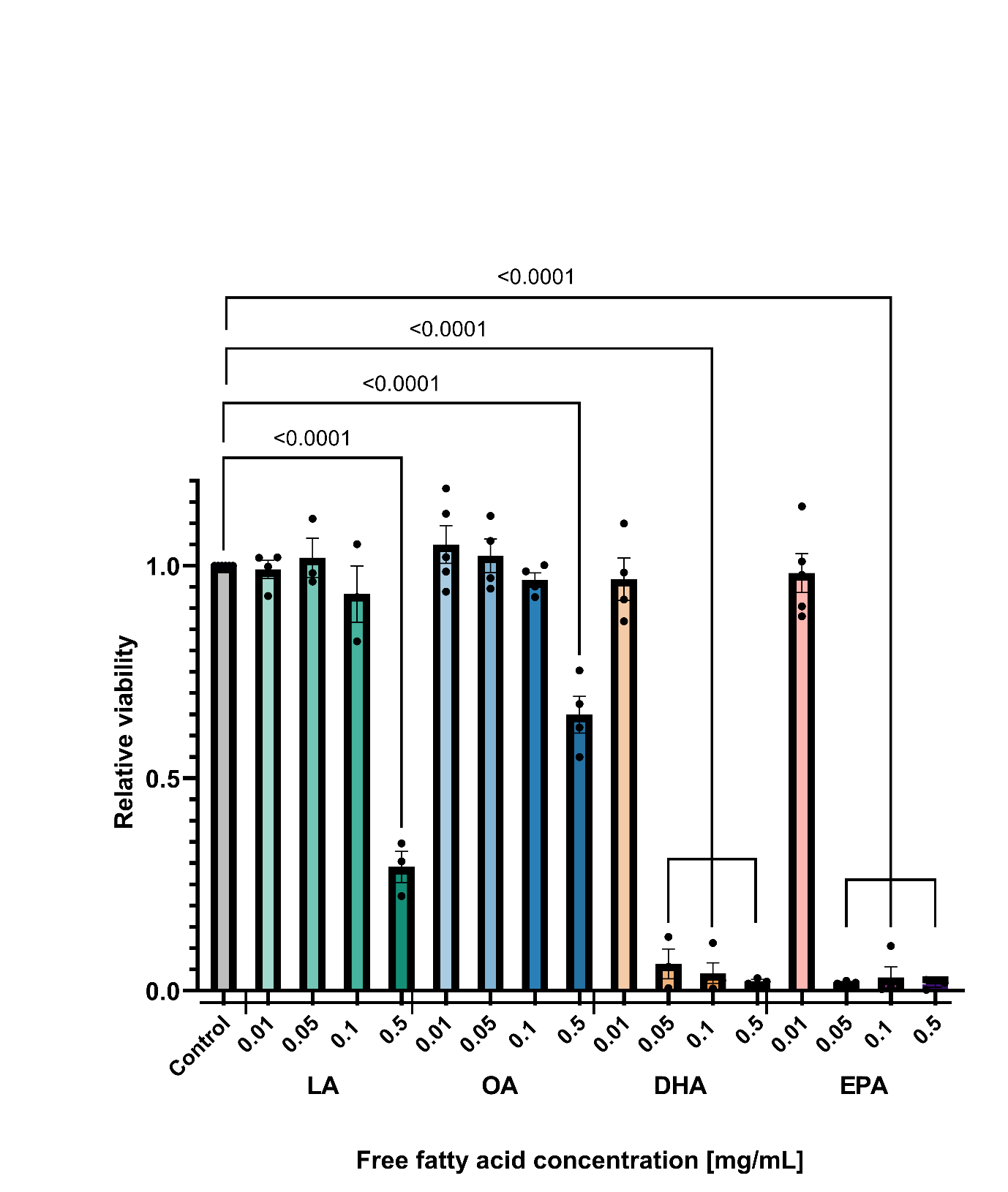 |
| --- |
| **Supplement Figure S4.** **Viability of Jurkat cells after 48-hour treatment with individual free fatty acids.** Jurkat cell viability after 48 h treatment with increasing concentrations (0.01–0.5 mg/mL) of free fatty acids: EPA, DHA (omega-3), LA (omega-6), and OA (omega-9). EPA and DHA caused a sharp viability drop, with nearly complete cell loss at 0.05 mg/mL. LA and OA showed lower toxicity, with significant effects only at 0.5 mg/mL; LA was slightly more toxic than OA. Relative viability was measured by MTS assay, calculated as the absorbance at 490 nm relative to the control. Each data point represents the average of 3 to 5 technical replicates. Bar plots show the mean ± SEM. Statistical significance was assessed using one-way ANOVA followed by Dunnett’s post hoc test, comparing all samples to the control. |


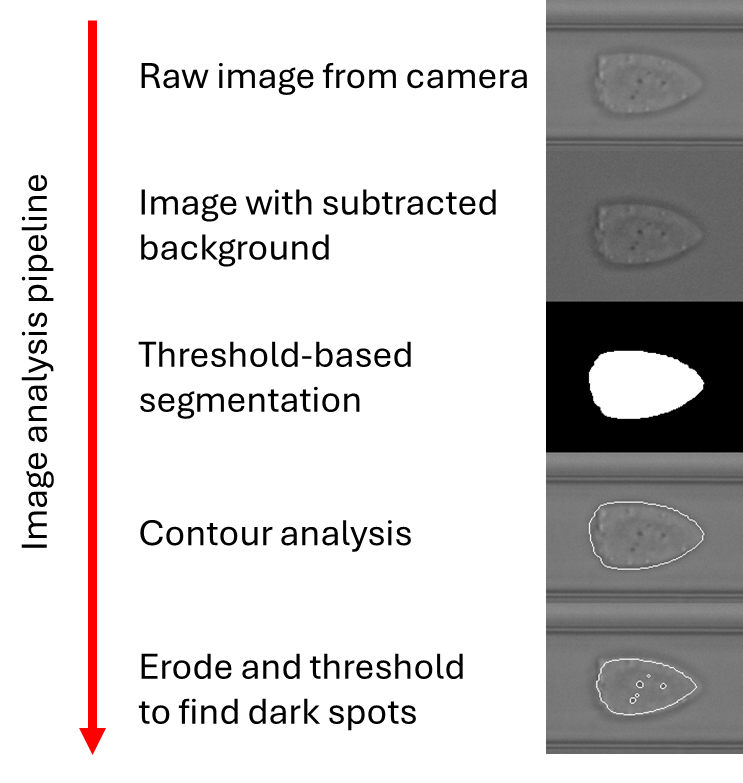


**Supplement Figure S5. Schematic representation of the image analysis pipeline** **for determining the amount of lipid droplets in a cell**.

First, the background image (image of the channel without cells) is subtracted from the raw cell image. Then, the image is segmented based on the adaptive threshold, and the cell contour is analyzed. To quantify the number of dark spots, the contour is first eroded, so that it encloses only the cell interior, and then the dark spots are quantified with adaptive thresholding. Algorithmic image analysis was implemented using standard Python libraries such as OpenCV and Pillow. The actual code is published at https://github.com/biophysics-ul/rtdc-toolbox.


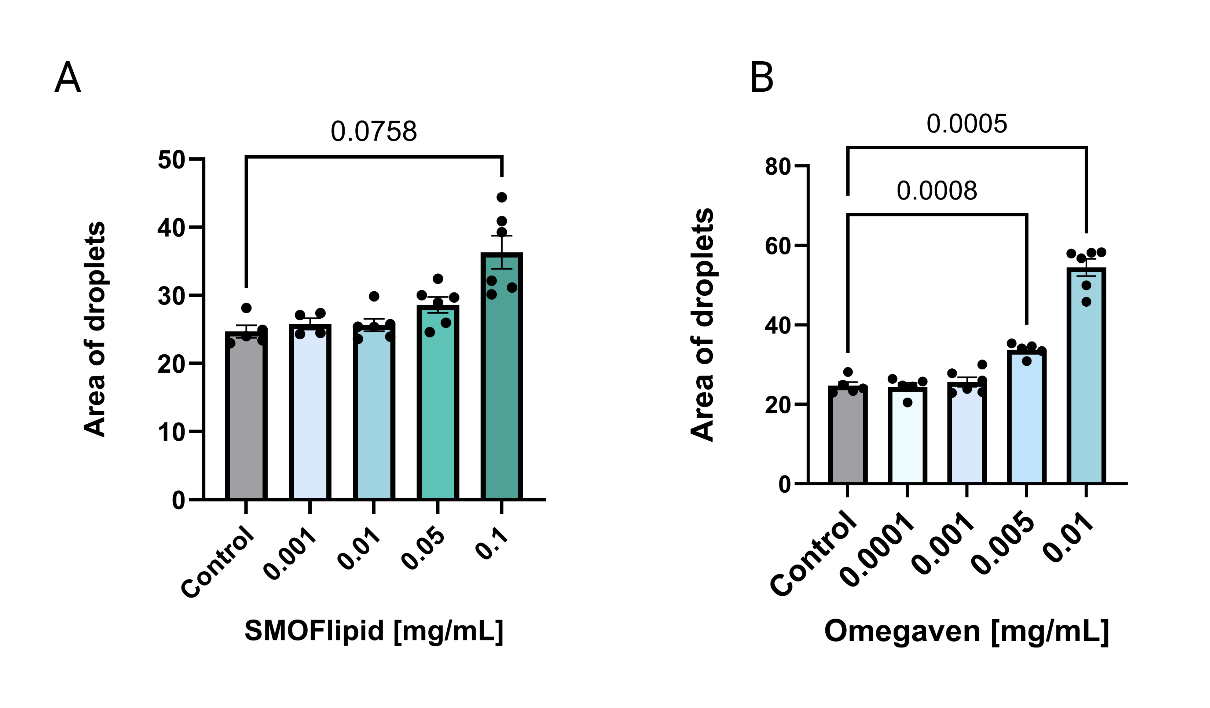


**Supplement Figure S6: Quantification of lipid droplet formation by area of droplets using DC image analysis.** Lipid droplet quantification can be performed in various ways, such as by counting the number of droplets (Figure 5) or measuring their total area (as shown here), both of which yield consistent trends.

A, B) Lipid droplet accumulation in Jurkat cells after 48 hours of incubation with varying concentrations of SMOFlipid (A) or Omegaven (B). Each data point on the graph represents the average area of lipid droplets measured in at least 900 cells from a single experiment. Bar plots show the mean ± SEM of these values, and bar colors indicate lipid emulsion concentrations. Statistical significance was assessed using mixed effects analysis followed by Dunnett’s post hoc test, comparing all samples to the control.

**Supplementary Figure S7: The effect of lipid emulsions on Jurkat cell ATP production.** Jurkat cells were treated for 48 h with SMOFlipid or Omegaven lipid emulsions at 0.1 mg/mL of total lipid emulsions for SMOFlpid and 0.01 mg/mL for Omegaven, after which the rate of oxygen consumption (OCR) and extracellular acidification rate (ECAR) were determined using a modified Seahorse Mito Stress Assay with a prior etomoxir injection to measure the contribution of beta oxidation. After 3 measurement cycles, 5 µM etomoxir or medium was injected, after which baseline OCR and ECAR were measured. 1.5 µM oligomycin, 1.5 µM carbonyl cyanide p-trifluoromethoxyphenylhydrazone (FCCP), and 0.5 µM rotenone + antimycin A (AA) were then sequentially injected to determine the ATP-synthase independent, maximal and non-mitochondrial OCR, respectively. The ATP production rate from oxidative phosphorylation (A) was calculated according to formula: OxPhosATP (pmol ATP/min) = (basal OCR – OCR after oligomycin) (pmol O2/min) * 2 (pmol O/pmol O2) * P/O (pmol ATP/pmol O2) assuming a P/O ratio of 2.75. Glycolytic ATP production (B) was calculated as glycolytic proton efflux rate according to equation: glycoATP Production Rate (pmol ATP/min) = glycoPER (pmol H+/min) = basalPER (pmol H+/min) – MitoPER (pmol H+/min) = basalPER – (basal OCR – OCR after rotenone/antimycin A) * 0.6. These two parameters were then used to calculate the OxPhos ATP to glycolysis ATP ratio (C) and total ATP production (D), the latter defined as the sum of the OxPhos and glycolysis ATP production. Data represent mean ± SEM of two independent experiments. No significant differences were found by two-way ANOVA with Dunnett’s post-hoc test.


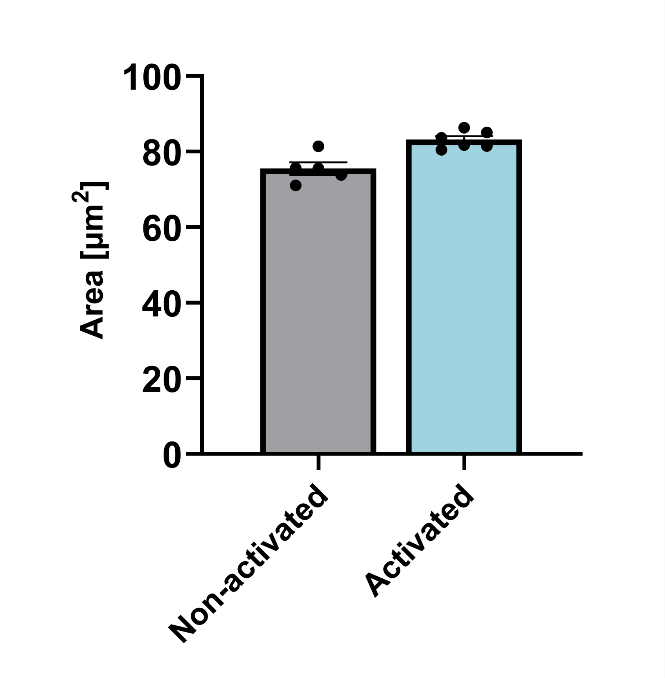


**Supplementary Figure S8. Comparison of cell size measured by deformability cytometry between non-activated and activated Jurkat cells.**

The projected cell area, as measured from deformability cytometry images, shows an approximate 10% increase in Jurkat cells after activation. This indicates a modest response compared to that typically observed in primary T cells. Each data point on the graph represents the mode of projected areas of at least 1,000 cells from one experiment. Bar plots show the mean ± SEM of these values.
